# Application of artificial intelligence to the public health education

**DOI:** 10.3389/fpubh.2022.1087174

**Published:** 2023-01-10

**Authors:** Xueyan Wang, Xiujing He, Jiawei Wei, Jianping Liu, Yuanxi Li, Xiaowei Liu

**Affiliations:** ^1^Laboratory of Integrative Medicine, Clinical Research Center for Breast, State Key Laboratory of Biotherapy, West China Hospital, Sichuan University, Chengdu, Sichuan, China; ^2^Research Center for Nano-Biomaterials, Analytical and Testing Center, Sichuan University, Chengdu, Sichuan, China; ^3^The First People's Hospital of Yibin, Yibin, Sichuan, China

**Keywords:** artificial intelligence, algorithm, big data, public health, education, curriculum

## Abstract

With the global outbreak of coronavirus disease 2019 (COVID-19), public health has received unprecedented attention. The cultivation of emergency and compound professionals is the general trend through public health education. However, current public health education is limited to traditional teaching models that struggle to balance theory and practice. Fortunately, the development of artificial intelligence (AI) has entered the stage of intelligent cognition. The introduction of AI in education has opened a new era of computer-assisted education, which brought new possibilities for teaching and learning in public health education. AI-based on big data not only provides abundant resources for public health research and management but also brings convenience for students to obtain public health data and information, which is conducive to the construction of introductory professional courses for students. In this review, we elaborated on the current status and limitations of public health education, summarized the application of AI in public health practice, and further proposed a framework for how to integrate AI into public health education curriculum. With the rapid technological advancements, we believe that AI will revolutionize the education paradigm of public health and help respond to public health emergencies.

## Introduction

The severe acute respiratory syndrome (SARS) outbreak in 2003 was the first epidemic that seriously impacted public health. In addition, H1N1 influenza, Ebola virus, polio, Zika virus, and coronavirus disease 2019 (COVID-19) have been declared public health emergencies of international concern by the World Health Organization, which have also led to the severe global public health crises (1, 2). Today, the COVID-19 pandemic and monkeypox outbreak in 2022 are still sweeping across the globe (3, 4), causing heavy burdens on healthcare, lives, and the world economy (5, 6). All of these reinforce the importance of strengthening public health systems. Public health workforce fighting on the front line is committed to disease control and provides effective health protection and health care for the health system. However, shortages of these professionals have severely hampered the implementation of preventive measures in this outbreak as expected, pointing to the demand to train more public health professionals through education (7, 8). Furthermore, the urgent needs of all sectors of society for public health emergency systems, vaccine development, and training of medical personnel have also highlighted the importance of medical education, especially public health education.

Recently, public health education remains largely based on traditional curricula with globally similar core competencies (9, 10). Public health education is generally based on five fundamental and core discipline areas including biostatistics, epidemiology, environmental health sciences, social and behavioral sciences, and health policy management (11, 12). To assess the educational content and educational competencies, interdisciplinary and cross-cutting subjects, such as communication and informatics, leadership, diversity and culture, professionalism, public health biology, program planning, and system thinking, are also incorporated into the public health curriculum of most schools (13). Moreover, core competencies for addressing public health practice challenges have also been incorporated into the certification criteria. Even so, a large part of the training process is focuses on absorbing much information as possible and learning how to apply that knowledge in practice, which is still based on memory (14). Additionally, under the traditional education model, the quality of teaching largely depends on the level of teachers. However, excellent teachers not only need long-term training but also are in short supply. Traditional coursework and book knowledge alone can hardly equip students to address real-world public health challenges (9, 14, 15). Teaching cannot keep up with new technologies and teaching content. Therefore, the optimization of public health education and the cultivation of public health professionals become the top priorities in the context of current epidemics (11, 16).

As an emerging technology in the twenty-first century, artificial intelligence (AI) has already penetrated various fields, including the economy, science, healthcare, and medicine, and has become an important driving force for their future development (17, 18). Nowadays, thousands of mature AI applications, such as virtual assistants, autonomous vehicles, facial recognition, and medical diagnosis, have been developed to solve problems in specific industries or institutions (19–21). AI-based financial applications such as Mint or Turbo Tax act as personal financial assistance, collecting personal data and providing sound financial advice (22, 23). Automakers such as Toyota, Volvo, and Tesla use AI techniques to train computers to think like humans to avoid accidents while driving in any environment (24). Facial recognition based on computer vision is the most common field of AI. Facial recognition technology on mobile phones and laptops can replace pin numbers and passwords, securing an individual's data (25). AI-based machine and deep learning models are being used to detect diseases such as skin, liver, heart, and Alzheimer's (26, 27). Among them, the recurrent neural network and the feed-forward neural network have achieved 97.59 and 100% in the diagnosis of liver disease hepatitis virus, respectively (28). Moreover, AI has been successfully applied to healthy education and clinical training, including ophthalmology (29), radiology (30), physical (31), and residency training (32). For example, Fang et al. (32) have applied AI-based pathologic myopia identification system in the ophthalmology residency training and achieved satisfactory training efficiency. All of these have shown the powerful potential of AI.

Notably, AI also plays a pivotal role in the transformation of education. Recently, AI has shown the potential to address educational challenges and innovate teaching-learning practices, enhancing teaching quality. AI-based intelligent tutoring systems that can identify and respond to students' knowledge gaps, personalized virtual tutors, two-way intelligent feedback between teachers and students, data mining, and the execution of mundane tasks will contribute to improving the quality of education (33). Here, we reviewed the fundamentals of AI, focused on the application of AI in public health practice, offered reasonable curriculum recommendations for introducing AI in public health education, and further discussed the prospect of AI in public health.

## Fundamentals of artificial intelligence

Broadly, AI refers to the process by which computers and machines simulate human behavior, including perception, learning, inferencing, analysis, and decision-making, to perform tasks through data processing and pattern recognition (34–36). AI includes multiple subsets or subfields, of which machine learning, deep learning, neural networks, computer vision, and robotics are the five main subfields of AI. Machine learning and deep learning are two major subfields that are often mentioned in AI (37, 38). Machine learning enables computers to learn autonomously by analyzing training data and experience without explicit programming. Furthermore, its performance improves with time. Machine learning algorithms include the supervised type that needs a training dataset containing input data and expected output and the unsupervised type that the data itself learns instead of the training datasets (39, 40). Recently, multiple complex problems in areas, such as finance, healthcare, and manufacturing, have been well-addressed through machine learning (41–43). Deep learning has aroused much concern due to its remarkable success in computer vision, speech recognition, and self-driving cars. Deep learning is not only another subset of AI but also a subset of machine learning. It belongs to a class of machine learning algorithms that use multiple layers of artificial neural networks as an architecture for data representation learning (40, 44–46). A deep neural network trained on more than 37,000 head computed tomography scans of intracranial hemorrhage evaluated 9,500 unseen cases, reducing the time to diagnose intracranial hemorrhage in new outpatient clinics by 96% with 84% accuracy (47). Neural networks are often used to create software that can imitate human learning and decision-making (46). Artificial neural networks composed of many interconnected processing nodes or neurons have the ability to learn to recognize patterns. Neural networks have shown enormous potential in improving decision-making in various industries and in improving the prediction accuracy of machine learning algorithms. The above subset is designed to make the computer think. However, computer vision enables computers and systems to take action or make recommendations after obtaining meaningful information from digital images, videos, and other visual inputs, enabling computers to see, observe and understand (48). Notably, the robotic system is an AI system deployed in physical form to control physical objects in the world through supervised and unsupervised learning (49). Up to now, several types of robotics systems have been developed to assist humans in difficult or dangerous tasks ranging from healthcare to national defense. All of these lay the foundation for further application and development of AI.

## The application of artificial intelligence in public health

Driven by major advances in computers algorithms and accumulation of big data over the decades, AI has entered an extraordinary stage of rapid development and widespread application. More recently, traditional AI research areas, including computer vision, speech recognition, and robotics, have also been found to be innovatively applicable in other real-world contexts, such as public health. In particular, the coronavirus pandemic outbreak at the end of 2019 plunged the world into a severe public health crisis. AI-based medical devices, algorithms, and other new industries have shown great potential in surveillance, prevention, diagnosis, and health management, which provides important support for this global fight against the epidemic.

### Disease surveillance and prevention

Disease surveillance in public health surveillance aims to detect early and reliable signals of health anomalies and epidemic outbreaks from the diverse collection of data sources. Data sourcing and analysis are two major challenges for public health surveillance. AI-based ubiquitous social media, online newswires, and other internet-based data streams effectively leverage various open data beyond traditional public health surveillance systems through its powerful surveillance capabilities, which expand and facilitate data sourcing (50). Traditional data analysis is primarily achieved through statistical methods that focus primarily on macro-level conclusions. However, as data complexity increases and data volumes accumulate, statistical inference becomes quite difficult within the framework of these methods. AI-based analytics methods such as natural language processing and image processing converted unstructured data into structured items through semantic labeling and auto-population of features, which in turn enhances traditional statistics-based data analysis (50). In practice, multiple AI products, such as infrared thermal imaging and face recognition, contribute to monitoring and controlling the source of infection. These products expressly identify people with abnormal body temperature and close contact to determine the source of the disease in the case of dense crowds and a rapid flow of people.

### Intelligent diagnosis

The most obvious manifestation of AI in public health is intelligent diagnosis. Intelligent diagnosis requires relevant personnel in medical institutions to collect and analyze a large amount of data and information by using modern information technology. Machine learning algorithms are then used to quickly identify the database of the cases to facilitate professionals making highly accurate diagnostic decisions (51). Recently, multiple auxiliary diagnostic AI devices have been approved by the U.S. Food and Drug Administration (FDA) for various diseases (52). IDx-DR is the first FDA-approved autonomous AI system that automatically analyzes retinal images for signs of diabetic retinopathy without the help of medical personnel (29). The clinical trial results showed that the accurate identification rates of IDx-DR for patients with more than mild diabetic retinopathy and those with less than mild diabetic retinopathy were 87.4 and 89.5%, respectively (53). Additionally, cancer diagnosis is one of the most important applications of AI-based intelligent diagnosis (54, 55). The algorithm developed based on deep learning was used to detect mammographic lesions with 99% accuracy, reducing unnecessary biopsies (56–59). Furthermore, colonoscopy videos can also be analyzed using AI to accurately identify polyps in real-time (60).

Excitingly, AI has shown potential in designing novel anticancer therapies or at least guiding the development of such therapies, which could reduce failure rates and shorten approval times (61, 62). The mechanism of anticancer molecules can be precisely predicted by AI algorithm BANDIT, leading to precise preclinical and clinical positioning, and increased likelihood of clinical success (63). Likewise, effective drug combinations can be predicted and screened based on AI tools, optimizing the treatment of cancer (64).

### Health administration

Additionally, AI technology can also improve the efficiency and quality of health management in various ways such as AI-assisted decision-making systems, medical record quality control, and pathological assistance systems. Health protection and promotion inseparable from AI. With advances in AI, personalized predictions and prevention are possible (65, 66). Up to now, multiple disease prediction models had been developed and improved to provide targeted and personalized health advice (67–70). Furthermore, AI has also greatly contributed to the development of the psychological counseling industry. Chatbots like Wysa ensure empathic support and advise on when to consult a human practitioner (71). Deep neural network-based AliveCor can detect possible heart rhythm abnormalities in users by evaluating heart rate data, physical activity and other influencing factors, reminding people to proactively manage their heart health (72, 73).

Taken together, AI has shown a profound impact on disease surveillance, intelligent diagnosis, and health management in public health systems, indicating that AI will strongly promote public health toward a more intelligent and humanized direction.

## The introduction of artificial intelligence in public health education

Public health education aims to equip students with sound theoretical understanding to meet complex health-related challenges with appropriate methods. Actually, almost all theoretical and practical issues related to health involve multidisciplinary collaboration (74, 75). However, only partial curriculums covered the necessity of interdisciplinarity in traditional public health education. In addition, theories, methods, health topics, and their applications are often taught side by side, making it difficult for students to combine them (74, 76). Fortunately, the application of AI in education brought new opportunities for health public education. The intelligence of public health education will provide guidance and technical support for training professionals in the new era, improving the efficiency and quality of education.

### The application of artificial intelligence in public health education

Recently, AI has already been applied in education, especially with some tools that help develop skills and test systems. As AI-based educational solutions continue to mature, AI is expected to help fill gaps in learning and teaching needs, enabling schools, and teachers to do more (77). Computer systems combined by incorporating human intelligence could serve intelligent tutors, tools, or students, facilitating decision-making in educational settings. Understanding individual differences is essential for developing teaching tools for specific students and tailoring education to individual needs at different stages. Identifying individual differences is essential for developing pedagogical methods for specific students and customizing education for individual needs at different stages (78). A large amount of personal data can be accurately collected by intelligent education systems based on big data and AI technologies. Learning patterns of students is revealed through data analysis to identify their unique preferences and experiences (79). Therefore, AI and big data have the potential to realize personalized learning and achieve precision education. Additionally, several basic activities in education, such as grading, frequently asked questions, and predicting learning outcomes, were automated by AI, which liberate teachers and encourage them to focus on professional development (80, 81). AI solutions can identify weaknesses by assessing the learning history of students to provide courses suitable for improvement. Notably, AI breaks down the barriers between schools and traditional grades and promotes the balance of teaching resources. Widespread application of virtual learning promotes the balance of teaching resources. Intelligent tutoring systems are an integral part of AI in education (82). These systems, such as AI chatbots, AI tutors, and tutoring programs, provide one-on-one instruction and feedback (83–85).

The application of AI technology in public health education focuses on the teaching of theoretical courses, which is in the innovation stage of educational informatization process. The traditional teaching pattern has been improved on the basis of computers and the Internet to achieve a certain degree of autonomous learning, collaborative learning, and teaching feedback. Notably, how to apply theoretical knowledge into practice is the focus of public health education beyond theoretical study. However, compared with other subjects that have gone beyond the theoretical stage, such as situational, inquiry-based and simulated medicine, there is a gap in the application of AI in public health education practice.

### Recommendations of the standardized curriculum

Objectives of the AI-based public health core course include recognizing major research and discoveries of AI in public health, and understanding learning potential applications in public health practice. The core curriculum should begin with basic mathematics and statistics lessons related to AI (86). Then, high-quality courses covering AI, machine learning, deep learning, and data science serve as the core curriculum of public health education, allowing students to focus on applications of these subjects more naturally in subsequent training. Finally, the curriculum should focus on how AI can be used in public health practice, such as disease prevention, infection source control, and health management (29). Specialized courses with extracurricular activities and dedicated training should also be considered for those students who wish to learn more. Additionally, refresher courses should also be provided for licensed professionals.

## Conclusions and prospects

With the increasing emphasis on public health education, the reform of teaching methods and the change of personnel training mode need to be accelerated. A new-era education system with interactive learning and intelligent learning as the main content urgently needs to be established. However, the existing facilities in medical colleges are difficult to achieve personalized education for students. The low education collaboration and the conflict between intelligent teaching and traditional teaching are not conducive to meeting the demand of society for higher-quality educational resources in public health education. The introduction of AI into education will open up novel opportunities to dramatically improve the quality of teaching and learning. On the one hand, AI enhances the personalization of student learning plans and lessons and facilitates tutoring by helping students improve their weaknesses and improve skills. On the other hand, educators could benefit from intelligent systems that facilitate assessment, data collection, and improved learning progress. Recently, AI has been introduced into various fields in education, such as ophthalmology (29), radiology (30), music (87), and physical (31), providing mature technical support and effectively promoting its development in a more intelligent and humanized direction. Therefore, with the help of education section, we could reform public health education and design formal integrated AI curriculum in medical schools to better equip public health professionals to respond to emergencies. It is believed that soon, public health education based on AI will inject unprecedented impetus into the development of the public health and improve the ability of public health system to respond to emergencies.

## Author contributions

All authors listed have made a substantial, direct, and intellectual contribution to the work and approved it for publication.
